# Supplementary material for: Quantum measurement arrow of time and fluctuation relations for measuring spin of ultracold atoms
Source: Nat Commun. 2021 Mar 23;12:1847. doi: 10.1038/s41467-021-22094-3 (PMC7988044; doi:10.1038/s41467-021-22094-3)
Supplement: Supplementary file 1 — Supplementary Information [file 41467_2021_22094_MOESM1_ESM.pdf]

Supplementary Information for "Quantum measurement arrow of time and fluctuation relations for measuring spin of ultracold atoms"

Maitreyi Jayaseelan,[1,2] Sreenath K. Manikandan,[1,2] Andrew N. Jordan,[1,2,3] and Nicholas P. Bigelow[1,2,*]

[1]*Department of Physics and Astronomy, University of Rochester, Rochester, NY 14627, USA*
[2]*Center for Coherence and Quantum Optics, University of Rochester, Rochester, NY 14627, USA*
[3]*Institute for Quantum Studies, Chapman University, Orange, CA, 92866, USA*

(Dated: February 12, 2021)

SUPPLEMENTARY NOTE 1: SUPPLEMENTARY METHODS

A. State preparation

The first step of our experimental protocol is to initialize our two-state quantum system into a quantum state

$$|\psi_0\rangle = c_1|\psi_\uparrow\rangle + c_{-1}|\psi_\downarrow\rangle. \tag{1}$$

We use a coherent two-photon Raman interaction to couple two ground states $|\psi_\uparrow\rangle$ and $|\psi_\downarrow\rangle$ of a three-level Λ system via an excited state $|e\rangle$ using optical fields with complex Rabi frequencies Ω_A and Ω_B (see Fig. 1 of the main text)[1]. Here we denote the initial atomic state for state preparation as $|\psi_i\rangle$. In the limit of large detuning Δ of the optical frequencies from the excited state, the dynamics is captured through the time evolution of the two-level system $\{|\psi_\uparrow\rangle, |\psi_\downarrow\rangle\}$ under the atom-field interaction as:

$$\partial_t|\psi\rangle = \frac{i}{4\Delta}\begin{pmatrix}|\Omega_A|^2 & \Omega_A^*\Omega_B \\ \Omega_A\Omega_B^* & |\Omega_B|^2\end{pmatrix}|\psi_i\rangle. \tag{2}$$

When the Raman interaction is effected with square diabatic pulses the right hand side contains terms that are constant in time so that the system of coupled equations can be integrated directly; for a time of interaction t, the evolution of the state vector then acquires the form

$$|\psi_f\rangle = e^{i\Omega t/2}\hat{\mathbf{U}}|\psi_i\rangle, \tag{3}$$

where $\hat{\mathbf{U}} = \exp\left(i\frac{\Omega t}{2}\mathbf{n}\cdot\boldsymbol{\sigma}\right)$ is the unitary time evolution operator [2, 3]. Here $\boldsymbol{\sigma}$ is the vector of Pauli matrices. The generalized Rabi frequency $\Omega = (|\Omega_A|^2 + |\Omega_B|^2)/4\Delta$, and the vector $\mathbf{n} = (\sin 2\alpha \sin\phi, \sin 2\alpha\cos\phi, \cos 2\alpha)^T$ in the unitary evolution are determined by experimental parameters: $\tan\alpha = |\Omega_A|/|\Omega_B|$ and $\phi = \phi_A - \phi_B$ describe the relative strengths and phase between the Raman beams in the experiment [2–4].

In the experiment the two-state quantum system is constituted by the $|F=2, m_F=2\rangle$ and $|F=2, m_F=0\rangle$ atomic Zeeman sublevels in the $|F=2\rangle$ ground state hyperfine manifold of ^{87}Rb. We create a Bose–Einstein condensate (BEC) in the $|\psi_i\rangle = |\psi_\uparrow\rangle \equiv |F=2, m_F=2\rangle$ atomic state in a magnetic trap [1–4]. The trap is turned off, and the cloud under free-fall expands for 9 ms to a regime where interatomic interactions are negligible and the dynamics can be described using the single-atom Hamiltonian of the atom-field interaction described above. A bias magnetic field of ≈ 10 Gauss defines a quantization axis for the system. The Raman pulses that prepare the initial quantum state in our experiment—two phase-coherent, diabatic, 5 μs square pulses derived from a single Raman master laser, and detuned 440 MHz below the excited state $|e\rangle \equiv |F'=1, m_F'=1\rangle$ on the D1 line of Rb—copropagate along this quantization axis and have polarizations that allow us to couple the $|\psi_\uparrow\rangle \equiv |F=2, m_F=2\rangle$ and $|\psi_\downarrow\rangle \equiv |F=2, m_F=0\rangle$ states via the excited state $|e\rangle$. The individual frequencies of the Raman pulses are controlled with acousto-optic modulators, so that the two atomic states, with a ground-state splitting of ≈ 7.2 MHz, are individually addressed by the optical fields.

For the results relating to the arrow of time and fluctuation relations for a spatially uniform preparation of the cloud, we use Gaussian Raman beams with uniform phase and orthogonal circular polarizations (σ_+, σ_-). The Raman beams are overlapped before being fiber-coupled so that they share the same Gaussian spatial mode and are essentially

* nbig@pas.rochester.edu

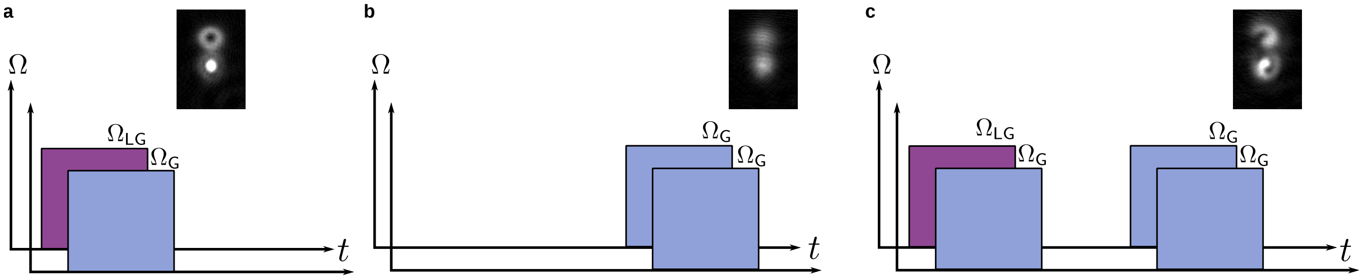

Supplementary Figure 1: Matter-wave interference verifies the spatial and spin coherence of the cloud at the time of imaging [1, 3, 4]. (a) shows a cloud created in an entangled superposition state as in Supplementary Equation 4, using a pulse pair with Laguerre–Gaussian (LG, with Rabi frequency $\Omega_{\rm LG}$) and Gaussian (G, with Rabi frequency $\Omega_{\rm G}$) Raman beams on an atomic cloud with population starting in $|F=2, m_F=2\rangle$. (b) shows the effect of a pulse pair using just Gaussian Raman beams on an atomic cloud with population starting in $|F=2, m_F=2\rangle$, showing transfer of $\approx 50\%$ of the atomic population between the spin states. (c) shows the resulting interference between the spin components of the cloud after the application of both pulse pairs to an atomic cloud starting in $|F=2, m_F=2\rangle$, the first preparing the entangled state of Supplementary Equation 4, and the second verifying the coherence through matter-wave interference. Persisting spatial and spin coherence at the time of imaging are apparent in the sinusoidally-varying intensity in each spin state in the resulting absorption image. Note that since the absorption imaging process is destructive, the three data shots shown are from separate experimental runs where the Raman process is performed each time on different clouds initially identically prepared in atomic state $|F=2, m_F=2\rangle$.

uniform in intensity over the spatial extent of the cloud ($\approx 50\,\mu$m at the time of the Raman interaction). This process thus controllably creates an ensemble of $\approx 6.5\times10^6$ identically prepared atoms in a coherent superposition state of the atomic ground states, which is the state $|\psi_0\rangle$ in our investigation of the quantum measurement process.

Images of the cloud are taken with standard absorption imaging techniques [5]. This obtains a column density of the cloud integrated along the quantization axis. The Stern–Gerlach interaction produces dynamics in the transverse plane, so that the density profile of the cloud in the transverse direction is a probability distribution for spin measurement with the Stern–Gerlach process. We note that since our image processing involves subtracting images taken at three successive times—we capture an absorption image of the atoms, a beam image without atoms, and a dark shot of background light, which we process to compute the optical density of the cloud—we see negative values in some pixels as a result of noise. The imaging system we use has unit magnification, and the camera has a pixel size of $16\,\mu$m $\times 16\,\mu$m.

In the subsequent results treating the fluctuation theorem in the context of the quantum many-body wavefunction of the BEC, we create an entangled superposition state of the cloud where a center-of-mass orbital angular momentum (OAM) quantum number ℓ is correlated with spin state. Here, one of the Raman beams is first sent through a spiral phase plate that creates a vortex Laguerre–Gauss mode with OAM quantum number $\ell=1$ and circular polarization σ_+. This beam is then overlapped with the second Raman beam in a Gaussian spatial mode and orthogonal circular polarization σ_- before interacting with the cloud, initially in state $|F=2, m_F=2\rangle$. The atoms that are transfered from $|F=2, m_F=2\rangle$ to $|F=2, m_F=0\rangle$ acquire an OAM equal to the difference of the OAM between the two Raman beams. This creates the entangled state,

$$|\psi_i\rangle = \alpha|LG\rangle|\psi'_\uparrow\rangle + \beta|G\rangle|\psi'_\downarrow\rangle, \tag{4}$$

where we define $|\psi'_\uparrow\rangle \equiv |F=2, m_F=0\rangle$ and $|\psi'_\downarrow\rangle \equiv |F=2, m_F=2\rangle$ for notational consistency with the analysis in Supplementary Note 3. Note that this assignment is opposite that used in the Gaussian analysis, which is the natural one, however the results discussed are unchanged by this since we are only concerned with relative attributes of the cloud as the spin states separate. The LG and G mode functions are further discussed in Supplementary Note 3.

We now address the important issue of relaxation and decoherence timescales in our system. The two-photon resonance linewidth of our Raman process is typically a few kHz. We use $5\,\mu$s pulses in our two-photon Raman transfer process to create superpositions of spin states, so that the time scales of state preparation are well within the time-scales of state decay. Quantum systems are inevitably coupled to their environment, and sources of homogenous and inhomogenous relaxation, including spin-spin interactions, and stray magnetic fields, are a concern. Here, we are benefitted by the ultracold temperatures of our dilute atomic cloud, and the excellent isolation from the environment, so that these sources of decoherence are well-controlled in our experiment. We demonstrate that the spatial and spin coherence of the cloud is retained at the time of absorption imaging, and is not destroyed during the free fall

of the cloud from the time of state preparation to the time of imaging. To do this, we consider again an atomic cloud prepared in a coherent superposition of spin states $|\psi'_\uparrow\rangle$ and $|\psi'_\downarrow\rangle$, with coupled orbital angular momenta ($\ell = 1$, $\ell = 0$) (Supplementary Fig. 1(a)). The state with angular momentum $\ell = 1$ contains an azimuthal phase that varies by 2π around the central axis of the mode, while the spin state with $\ell = 0$ has a uniform phase. We then apply a second $5\,\mu$s pulse pair with effectively uniform Gaussian profiles and angular momentum quantum numbers ($\ell = 0$, $\ell = 0$), that transfers $\approx 50\%$ of the atomic population between each state (Supplementary Fig. 1(b) shows the atomic state prepared by just this second pulse pair, with no preceding LG-G transfer). Supplementary Fig. 1(c) shows an example of the absorption image produced by an atomic cloud after such a sequence when first an LG-G pulse pair and subsequently a G-G pulse pair are applied, showing matter-wave interference between the coherent superposition states [1, 3, 4]. The azimuthal phase winding from the $\ell = 1$ component produces a sinusoidal intensity variation in the absorption image of each spin state, and indicates the robustness of the atomic coherences written into the macroscopic BEC wavefunction. Further the absorption image indicates that this spatial and spin coherence is not destroyed on expansion of the cloud from the time of the Raman state preparation up to the time of absorption imaging. This coherence is still visible when the relative time delay of the second (interfering) pulse pair from the first (state preparation) pulse pair is varied; in the data shown in Supplementary Fig. 1(c) the time between the two pulse pairs was $10\,\mu$s, the measurement time $\tau = 1100\,\mu$s, and the image was taken after the time-of-flight of 13 ms. We can thus treat our atomic spin state as coherent over the timescales of the experiment.

B. Time-of-flight Stern–Gerlach absorption imaging

Our weak measurement of spin state relies on a readout provided by a time-of-flight Stern–Gerlach process that correlates atomic spin state with position. In examining the evolution of our initial atomic state during this process, we note that the homogeneous acceleration of the atomic wave-packet due to free-fall under gravity produces a center-of-mass dynamics that may be separated out from dynamics that affects the relative attributes of the atomic spin states (see for example analysis in [6]); this center-of-mass dynamics is excluded from our subsequent analysis. Further, we model the Stern–Gerlach interaction as a momentum kick, since the Stern–Gerlach magnetic field is pulsed on for a time τ much shorter than the subsequent free evolution time t_f. The relative kinetic energy of the components of the cloud at this stage of our experiment is small enough that to a first approximation we ignore evolution from the free Hamiltonian during the Stern–Gerlach interaction. We thus model our measurement dynamics in two stages: a stage where the dynamics is dominated by the interaction with the inhomogenous Stern–Gerlach magnetic field with interaction Hamiltonian H_{SG}, followed by a stage where the dynamics is governed by the free Hamiltonian $\hat{H}_{\text{free-fall}}$.

The Stern–Gerlach interaction is a magnetic dipole interaction between magnetic field and atomic spin:

$$\hat{H}_{\mathrm{SG}} = -\boldsymbol{\mu}\cdot\mathbf{B} = -g_F\mu_B\boldsymbol{\sigma}\cdot\mathbf{B} \tag{5}$$

where g_F is the Landé factor and μ_B the Bohr magneton. Here, we have a magnetic field that maintains the quantization axis in the z direction but has a gradient $B_0 \approx 300\ \mathrm{G\,cm^{-1}}$ in the x direction. This inhomogeneous field is pulsed on for a short time τ so that we may neglect evolution from the free Hamiltonian, and find the unitary evolution operator for the atomic state to be

$$\hat{\boldsymbol{\mathcal{U}}}_{\mathrm{SG}} = \exp\left(-i\delta p_x\hat{\sigma}_z\otimes\hat{x}/\hbar\right) \tag{6}$$

where $\delta p_x = g_F\mu_B B_0\tau$.

Our measurement process begins with our cloud in a coherent superposition of spin states $|s\rangle$, that are here expressed as eigenstates of $\hat{\sigma}_z$ with eigenvalues $s = \pm 1$. At this stage the quantum state is a product state of spatial and spin components $|\psi_0\rangle \equiv |\alpha\rangle|s\rangle$. In the Gaussian approximation we can write our initial position space wavefunction as a Gaussian wavepacket centered on $x = 0$ with width σ:

$$\psi_\alpha(x,0) = \langle x|\alpha\rangle = \frac{1}{\sqrt[4]{2\pi\sigma^2}}e^{-x^2/4\sigma^2}. \tag{7}$$

The wavefunction for atoms in spin state $|s\rangle$ after the Stern–Gerlach momentum kick from the magnetic field is

$$\psi_\alpha(x,\tau) = \langle x|\hat{\boldsymbol{\mathcal{U}}}_{\mathrm{SG}}|\alpha\rangle = \frac{1}{\sqrt[4]{2\pi\sigma^2}}e^{-x^2/4\sigma^2 + i\delta p_x sx/\hbar}. \tag{8}$$

This wavepacket shows dispersion through evolution under the free Hamiltonian $\hat{H}_{\text{free-fall}} = \hat{p}^2/2M$ where M is the atomic mass. Starting from the initial preparation for the pseudo-spin, $|s_0\rangle = c_1|\psi_\uparrow\rangle + c_{-1}|\psi_\downarrow\rangle$, an evolution time t_f

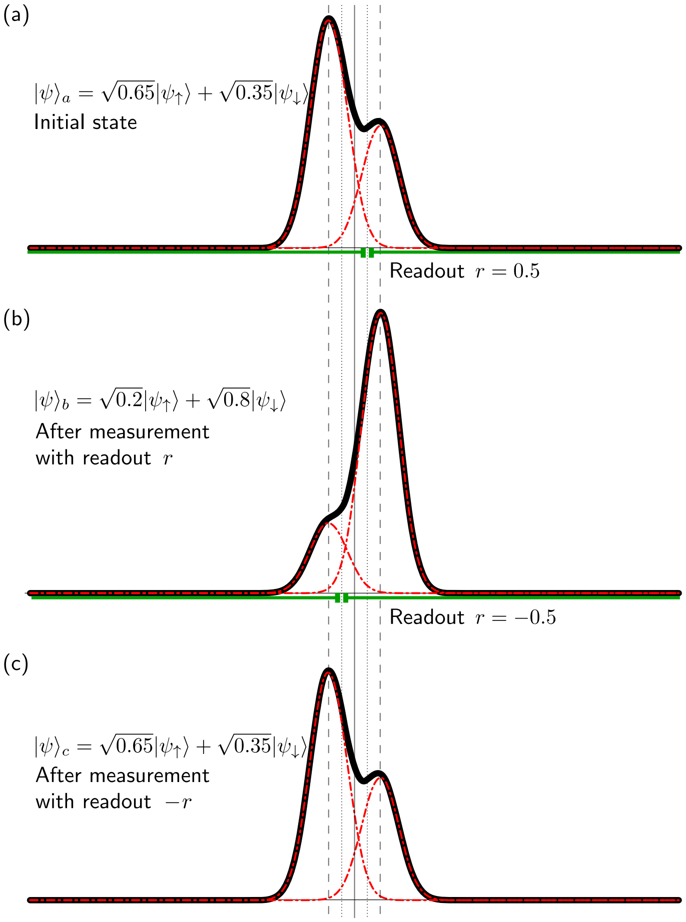

Supplementary Figure 2: An example of successful measurement reversal in the weak measurement of spin state where the measurement probabilities are Gaussian functions centered at ± 1. (a) A measurement on the initial spin state yields result r with a probability determined by the distribution. (b) The new spin state after measurement defines an updated probability distribution for readout, and shows here a finite probability for a subsequent measurement to yield readout $-r$. (c) The spin state after the second measurement shows a successful measurement reversal for this case.

in free fall leaves us with the probability distribution in position space,

$$|\psi(x,t_f)|^2 = \frac{|c_1|^2}{\sqrt{2\pi\sigma^2|\beta|^2}} \exp\left[-(x-\delta p_x t_f/M)^2/(2\sigma^2|\beta|^2)\right] + \frac{|c_{-1}|^2}{\sqrt{2\pi\sigma^2|\beta|^2}} \exp\left[-(x+\delta p_x t_f/M)^2/(2\sigma^2|\beta|^2)\right], \quad (9)$$

where $\beta = (1 + i\hbar t_f/2M\sigma^2)$. The two different spin states evolve as Gaussian wavepackets centered at $\pm\delta p_x t_f/M$ with a scaled width $\sigma|\beta|$, justifying our use of Gaussian Kraus operators in our analysis.

SUPPLEMENTARY NOTE 2: AN EXAMPLE OF MEASUREMENT REVERSAL

As an example of measurement reversal in a succession of weak measurements of spin state with a Stern-Gerlach apparatus, consider the sequence depicted in Supplementary Fig. 2. An atomic cloud is prepared in a coherent superposition of spin states $|\psi\rangle_a = \sqrt{1-a}|\psi_\downarrow\rangle + \sqrt{a}|\psi_\uparrow\rangle$. For a time-of-flight Stern–Gerlach process, we can expect the spin state to be correlated with position so that the probability distribution for our readout is the sum of two Gaussians; our distributions may be shifted and rescaled to give (for $\sigma^2 = 1/2$),

$$|\psi(r)|^2 \propto a \exp\left[-(r-1)^2\right] + (1-a)\exp\left[-(r+1)^2\right].$$

Suppose a weak measurement of spin state for the state $|\psi\rangle_a$, gives a readout $r = 0.5$. We can now use quantum Bayesian approach to infer that the spin state is then $|\psi\rangle_b$. Suppose we could select out this portion of the cloud with

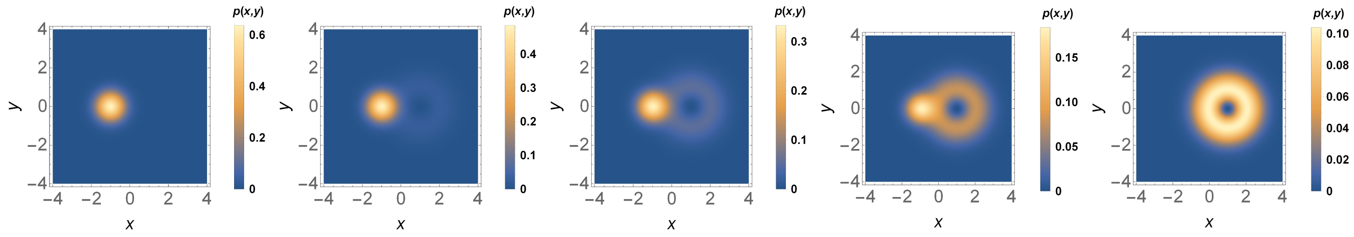

Supplementary Figure 3: Separated cloud for different z, the initial state of the qubit. The panels represent $z = -1$ to $z = 1$ in increment of $dz = 0.5$. We assume $\sigma_x = 0.5,\ \sigma_y = 0.5,\ w = 1.5,\ x_{0G} = -1,\ x_{0LG} = 1,\ y_{0G} = 0$, and $y_{0LG} = 0$.

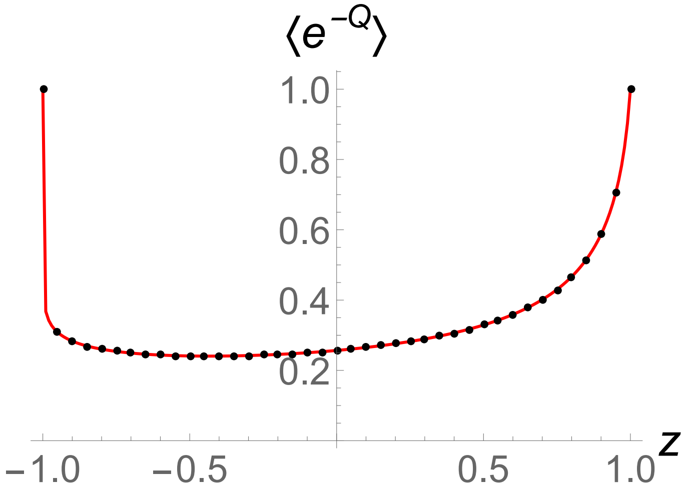

Supplementary Figure 4: Verifying fluctuation theorem for different initial spin state, denoted by their spin expectation value, $z = \langle\psi_i'|\hat{\sigma}_z|\psi_i'\rangle$, where $\hat{\sigma}_z$ is the Pauli spin matrix. We assume $\sigma_x = 0.5,\ \sigma_y = 0.5,\ w = 1.5,\ x_{0G} = -1,\ x_{0LG} = 1,\ y_{0G} = 0$, and $y_{0LG} = 0$. The dots indicate μ computed independently. The asymmetry in this curve follows from the asymmetry between LG and G modes.

a second slit, and perform a second Stern–Gerlach weak measurement on it. If our second measurement on this spin state now yields readout $-r$, or equivalently $r = -0.5$—which can happen with a finite probability as determined by the probability distribution in panel (b)—we must infer that the spin state is $|\psi\rangle_c$ with the associated probability distribution; but this is just the original spin state $|\psi\rangle_a$. This is an example of successful measurement reversal.

SUPPLEMENTARY NOTE 3: A QUANTUM MANY-BODY FLUCTUATION THEOREM FOR QUANTUM SPIN MEASUREMENTS USING A BOSE–EINSTEIN CONDENSATE

We verify a quantum fluctuation theorem which describes the quantum many-body dynamics of a Bose–Einstein condensate (BEC) subject to weak quantum spin measurements. A quantum coherent cloud of atoms is treated as a logical qubit in a designated spin state, where the quantum spin information is encoded spatially across the cloud. The logical qubit subsequently undergoes a quantum spin measurement via an absorption imaging technique that projects the collective atomic wavefunction into the position basis, yielding a many-body statistics for the quantum measurement process, with associated spin states inferred from the quantum Bayesian update rule. For this analysis, we define an atomic cloud qubit in the initial state,

$$|\psi_i'\rangle = \alpha|LG\rangle|\psi_\uparrow'\rangle + \beta|G\rangle|\psi_\downarrow'\rangle = \alpha|\Uparrow\rangle + \beta|\Downarrow\rangle, \tag{10}$$

where the spin information is encoded spatially across the many-body quantum system as,

$$|\Uparrow\rangle = |LG\rangle|\psi_\uparrow'\rangle \quad \text{and} \quad |\Downarrow\rangle = |G\rangle|\psi_\downarrow'\rangle.$$

The state $|\psi_i'\rangle$ represents the quantum coherent state of the atomic cloud just prior to the absorption imaging, which is really the act of quantum measurement in our experiment. Here LG and G represent the following Laguerre–Gaussian (with associated azimuthal and radial quantum numbers $\ell = 1, p = 0$), and Gaussian ($\ell = 0, p = 0$) spatial modes at the imaging plane,

$$\langle x,y|LG\rangle = \frac{2\sqrt{(x-x_{0LG})^2+(y-y_{0LG})^2}}{\sqrt{\pi}w^2} e^{-w^{-2}(x-x_{0LG})^2-w^{-2}(y-y_{0LG})^2} e^{i\phi(x,y)},$$
$$\langle x,y|G\rangle = \frac{1}{\sqrt{2\pi\sigma_x\sigma_y}} e^{-(4\sigma_x)^{-2}(x-x_{0G})^2-(4\sigma_y)^{-2}(y-y_{0G})^2}. \tag{11}$$

where $(x_{0LG},\ y_{0LG}), (\ x_{0G},\ y_{0G})$ are coordinates representing the centers of LG and G modes, σ_x, σ_y are the Gaussian standard deviations, and w is the waist of the Laguerre–Gaussian mode. We absorb all the associated phases of the Laguerre-Gaussian mode to the real function $\phi(x,y)$. We have kept the center of the LG and G mode functions arbitrary in order to account for the possibility that a magnetic field gradient may separate them relative to each other, conditioned on the spin state $|\psi_\uparrow'\rangle$ or $|\psi_\downarrow'\rangle$. See Supplementary Fig. 3. Now a two dimensional imaging of the cloud produces the following local quantum spin state update which can be thought of as the update of the logical qubit state $|\psi_i'\rangle$,

$$|\psi_f(x,y)\rangle \propto \langle x,y|\psi_i'\rangle = \hat{M}_F(x,y)\big(\alpha|\psi_\uparrow'\rangle + \beta|\psi_\downarrow'\rangle\big) = \hat{M}_F(x,y)|\psi_i''\rangle, \tag{12}$$

where the quantum state $|\psi_i''\rangle = \alpha|\psi_\uparrow'\rangle + \beta|\psi_\downarrow'\rangle$ encodes the same quantum information as encoded in the initial logical qubit state $|\psi_i'\rangle = \alpha|\Uparrow\rangle + \beta|\Downarrow\rangle$. This allows us to characterize the irreversibility of quantum measurement process in the experiment by considering the information dynamics resulting from the quantum measurement (cloud absorption imaging), in terms of the mapping between qubit states expressed in Supplementary Equation (12). The measurement operator $\hat{M}_F$ is defined as,

$$\hat{M}_F(x,y) = \begin{bmatrix} LG(x,y) & 0 \\ 0 & G(x,y) \end{bmatrix}. \tag{13}$$

The forward probability density $p_F(x,y)$ corresponding to the measurement process is,

$$p_F(x,y) = \langle\psi_i''|\hat{M}_F^\dagger(x,y)\hat{M}_F(x,y)|\psi_i''\rangle = |\alpha|^2|LG(x,y)|^2 + |\beta|^2|G(x,y)|^2. \tag{14}$$

This probability distribution $p_F(x,y)$ is directly measured in the experiment. Similarly, the measurement is reversed by the following measurement operator [7, 8],

$$\hat{M}_B(x,y) = \theta^{-1}\hat{M}_F^\dagger(x,y)\theta = \begin{bmatrix} G(x,y) & 0 \\ 0 & LG(x,y) \end{bmatrix}, \tag{15}$$

where θ is the time-reversal operator for a qubit. We obtain the associated backward probability density, $p_B(x,y) = ||\hat{M}_B(x,y)\hat{M}_F(x,y)|\psi_i''\rangle||^2/p_F(x,y) = |LG(x,y)|^2|G(x,y)|^2/p_F(x,y)$ only defined at points where the forward probability density $p_F(x,y)$ is nonvanishing. Given the forward and backward probabilities, we define the statistical arrow of time for the quantum measurement process as [7–9],

$$Q(x,y) = \log\frac{p_F(x,y)}{p_B(x,y)} = \log\frac{p_F^2(x,y)}{|LG(x,y)|^2|G(x,y)|^2}. \tag{16}$$

Note that the relative phase between LG and G modes does not appear in $Q(x,y)$. The arrow of time $Q(x,y)$ as a function of position of the atoms in the final cloud absorption image can therefore be determined from the experimental data $p_F(x,y)$, and the fit parameters for the LG and G functions obtained from the experimental data. We can now use this to state the fluctuation theorem for quantum measurements for the quantum many-body context discussed above as,

$$\big\langle e^{-Q}\big\rangle = \int dx\,dy\,p_F(x,y)e^{-Q(x,y)} = \int dx\,dy\,p_B(x,y) = 1-\mu, \tag{17}$$

where

$$\mu = \int dx\,dy\,\frac{|\langle\bar{\psi}_i''|\hat{M}_F^\dagger(x,y)\hat{M}_F(x,y)|\psi_i''\rangle|^2}{p_F(x,y)}, \tag{18}$$

and $|\bar{\psi}_i''\rangle$ is the qubit state orthogonal to $|\psi_i''\rangle$ [7]. See Supplementary Fig. 4 where we verify the fluctuation theorem using numerical simulations.

In the experiment, this cloud is prepared using a coherent two-photon Raman technique that associates the spin and orbital degrees of freedom of the atomic system by using beams containing orbital angular momentum (see state preparation Supplementary Note A). The modes of these Raman beams are Gaussian ($\ell = 0$) and Laguerre–Gaussian ($\ell = 1$) modes. The cloud begins in state $|\psi_\downarrow'\rangle$ in a Gaussian spatial mode, and atoms that are transferred to state $|\psi_\uparrow'\rangle$ pick up a vortex phase from the Raman beams, associating the LG and G mode functions with the spin states. Note that these are not true LG and G spatial modes of the cloud in the experiment, since the true spatial mode of the cloud depends on the complex interaction with the optical fields [3]; however to a first approximation the spatial modes follow the characteristic shapes of these functions. To obtain a better fit to the experimental data we empirically assign a mode function to the Gaussian portion of the cloud that is a bimodal Gaussian distribution with different variances (σ_x, σ_y) and $(\sigma_{x_0}, \sigma_{y_0})$, co-centered on $(x_{0G},\, y_{0G})$. In place of the Gaussian density $|G(x, y)|^2$, we then use a modified density,

$$|H(x,y)|^2 = \frac{d}{2\pi\sigma_x\sigma_y} e^{-(2\sigma_x)^{-2}(x-x_{0G})^2-(2\sigma_y)^{-2}(y-y_{0G})^2} + \frac{1-d}{2\pi\sigma_{x_0}\sigma_{y_0}} e^{-(2\sigma_{x_0})^{-2}(x_0-x_{0G})^2-(2\sigma_y)^{-2}(y_0-y_{0G})^2}, \tag{19}$$

where the parameter d weights the bimodal distribution while ensuring overall normalization when included with the Laguerre–Gaussian portion of the cloud. The form of the effect matrix–$\hat{M}_F^\dagger(x, y)\hat{M}_F(x, y)$–used in the fluctuation theorem results uses this modified density. The association of the orbital angular momentum quantum number with spin state is unaffected by this change, as also the essential results of the fluctuation theorem. The Laguerre–Gaussian density function used is the standard form

$$|LG(x,y)|^2 = \frac{4}{\pi w_x w_y}\left(\frac{(x-x_{0LG})^2}{w_x^2} + \frac{(y-y_{0LG})^2}{w_y^2}\right) e^{-2w_x^{-2}(x-x_{0LG})^2-2w_y^{-2}(y-y_{0LG})^2}. \tag{20}$$

In comparison, note that the effect matrix in the Gaussian–Gaussian Stern-Gerlach experiment for uniformly spin initialized atomic cloud has the following form: $\hat{M}^\dagger(r)\hat{M}(r) = (2\pi\sigma^2)^{-\frac{1}{2}}\exp[-(r-\hat{\sigma}_z)^2/(2\sigma^2)]$, where r is the scaled position readout variable as defined in the main text.

SUPPLEMENTARY NOTE 4: ADDITIONAL DATA USED IN THE MANUSCRIPT

C. Additional data for $\sigma^2 \simeq 0.35$ when cloud is uniformly initialized

Here we provide additional data from processing for Figure 2 of the Main text, detailing how we construct the probability distributions used in the manuscript.

We construct arrow of time distributions for different initial spin states that are measured with the same measurement strength set by σ^2 in Supplementary Figs. 5–16. The absorption image of the cloud is used to obtain a lineout through the median of the cloud passing through the centers of the two Gaussian wavepackets associated with the two spin states. In order to reduce noise we construct an average lineout using the lineout through the median of the cloud and the nearest neighbouring column on each side [shown in panel (a)]. We then fit our lineout to a two mode Gaussian profile [shown in panel (b)], while negative values from the raw data are omitted in the fit by making the ansatz that they come from background noise. The distribution is re-scaled and displaced to correspond to spin eigenvalues, ± 1 [shown in panel (c)]. This fit is used in subsequent analysis to construct our theory predictions for the fluctuation theorem and arrow of time distribution [shown in panel (d)]. The arrow of time distribution requires the forward and backward probabilities for a given readout r. This is constructed by first noting that the forward probabilities are merely given by the probability distribution determined by the two-mode Gaussian profile of the cloud. The corresponding backward probability requires, in addition, information about the standard deviation of individual Gaussians, obtained from the Gaussian fit.

D. Additional data for $z \simeq 0$ when cloud is uniformly initialized

Here we provide similar additional data from processing for Figure 2 of the Main text (Supplementary Figs. 17-24), detailing how we construct the probability distributions used in the manuscript.

E. Additional data for LG-G entangled initialization of the cloud

Here we provide additional data used in the discussion of the quantum many-body fluctuation theorem. The cloud is prepared in an entangled superposition state

$$|\psi_i'\rangle = \alpha|\Uparrow\rangle + \beta|\Downarrow\rangle \tag{21}$$

that is the initial state of our analysis. The data in Supplementary Figs. 25-36 show absorption images of the atomic cloud after a weak Stern–Gerlach measurement. Here again, since the absorption images are processed to obtain the cloud column densities, small negative values occur due to noise. These pixels are not used in the calculations of experimentally determined quantities. We fit the mode functions of Supplementary Equations (19) and (20) to the positive-valued absorption image to obtain fit parameters for the cloud to be used in our model. The irreversibility is computed from data and from theory using the fit parameters. Supplementary Fig. 41 shows the values for $\langle e^{-Q}\rangle$ and irreversibility parameter μ, where the values plotted are (black dots) $\langle e^{-Q}\rangle$ and (brown dots) $1-\mu$ as computed using the experimental data. Also shown are the (blue diamonds) theoretically calculated values for $\langle e^{-Q}\rangle$.

SUPPLEMENTARY NOTE 5: ERROR ANALYSIS OF THE FLUCTUATION THEOREM

The fluctuation theorem is numerically verified from single shot realizations of the experiment, and therefore each data point in Figures 2(b,d) and Figure 3 of the main text corresponds to a realization of the experiment. The cloud absorption imaging technique provides the entire statistics in a single shot from which we extract the experimental forward probability density (directly proportional to the measured intensity) as well as the Gaussian and Laguerre–Gaussian fit parameters, which is sufficient to compute the arrow of time Q from the experiment (see Equation (2) of the main text). As the number of counts recorded by the CCD camera is very large, the errors in estimating the LHS and RHS of the fluctuation theorem from the experimental data using this single shot imaging scheme do not originate from having fewer realizations of the experiment, but rather arise from imperfections in the theoretical modeling, as well as the imaging process itself; for instance, we fit the measured data for the experiment to a weighted sum of two identical Gaussians in the first example, but note that the spatial mode of the BEC wavefunction is not a true Gaussian, and only tends to Gaussian form after expansion. Additional systematic errors arising from the absorption imaging process include saturation effects and noise due to background variations during the imaging sequence. Error from saturation in the data are visible at the peak of some of the measured distributions, and the Gaussian approximation provides a reliable—as can be observed from the accuracy of the fit to data points near the peak—extrapolation for the peak intensity value in these cases. These errors do not vanish if the experiment is repeated a large number of times as they arise due to imperfections in our model/imaging process itself, and would tend to zero only for the case when the theoretical fit to the data is perfect. We identify three sources of error in our analysis which are treated as independent so the relative errors are added in computing the error bars provided in Figures 2(b,d) and Figure 3 of the main text.

The first source of error we considered includes the systematic error arising due to renormalization of measured positive intensities. To account for this, we compute the relative error we make in simulating an ideal experiment, where we renormalize the simulated distribution after removing the corresponding data points which shows negative intensity due to background noise in the experimental data. We compute the $\langle e^{-Q}\rangle$ for both simulated data sets and compute the relative error w.r.t the simulated experiment where those data points not accounted for in the experiment are correspondingly omitted for the simulation. This gives an estimate for the relative error RE(norm) from renormalization of measured positive intensities in an ideal experiment, and we get the estimate for the contribution to error bar in $\langle e^{-Q}\rangle_{\text{exp}}$ for the experimental data by multiplying $\text{RE(norm)}\times\langle e^{-Q}\rangle_{\text{exp}}$.

With the renormalized experimental data, next we estimate the error arising from imperfect fit to our theory model in the following manner. We can re-write the LHS of the fluctuation theorem as,

$$\langle e^{-Q}\rangle = \int \mathrm{d}r\, p_B(r) \approx \sum_i p_B(r_i)\mathrm{d}r_i \approx dr \sum_i y_i, \quad \text{where} \quad y_i = p_B(r_i), \tag{22}$$

and $p_B(r_i)\mathrm{d}r_i$ is the backward probability density around the discrete (binned) readout r_i having binwidth $\mathrm{d}r_i \approx \mathrm{d}r$, a constant determined by the pixel size and scaling we choose. We partially account for the error due to the negative intensity points which are excluded in our analysis as the error in estimating $\mathrm{d}r$. We estimate the relative error in $\mathrm{d}r$ as, $\text{RE}(\mathrm{d}r) = \mathrm{d}r^{-1}(|\max(r) - \min(r)|/(\text{number of bins}) - \mathrm{d}r)$. A similar approach is taken for the LG-G data, where we replace $\mathrm{d}r$ with $\mathrm{d}A$, the two dimensional area element, and the relative error in this area element is similarly estimated from the area missing from excluding the negative intensity points. To this, we add the relative error from

the sum of backward probabilities shown in Supplementary Equation (22) to estimate the contribution to the error in our estimate for $\langle e^{-Q}\rangle$ from irregularities in the image taken. This is computed in the following manner. Each of the data points y_i can be computed from the experimental data, as well as from the theory fit we use, giving the theory prediction y_i^0 for each i. See Supplementary Fig. 37(c) where the points y_i and y_i^0 are shown for one example. A difficulty is that because the experimental data we use is from a single shot, the error bar σ_i for each of the data points y_i is not provided. Nevertheless, we can estimate this error bar from the χ^2 statistics,

$$\chi^2 = \sum_{i=1}^{N} \frac{(y_i - y_i^0)^2}{\sigma_i^2}. \tag{23}$$

In order to do so, we make the following assumptions: (1) each of the σ_i are identical and equal to σ_0, and (2) χ^2 evaluated per degree of freedom is equal to one. This is equivalent to assuming that if we were to repeat the experiment many times, each of the data points y_i are distributed as a Gaussian with approximately the same width. Such an approximation for σ_0 when σ_i is not available is typically used to estimate the errors in the fit parameters (see for instance Appendix D of [10]). We therefore have,

$$1 = \frac{1}{M}\sum_{i=1}^{N} \frac{(y_i - y_i^0)^2}{\sigma_0^2} \implies \sigma_0^2 = \frac{1}{M}\sum_{i=1}^{N}(y_i - y_i^0)^2. \tag{24}$$

We denote the number of degrees of freedom $M = N - P$, where P is the number of constraints which relate the data points y_i. The number of constraints P can be approximated by the number of fit parameters we use in the problem, which determine the relations between data points y_i.

Now σ_0 is the error associated to each y_i, but we are interested in providing error bars $\pm\sigma_{\text{LHS}}$ for the sum, $\langle e^{-Q}\rangle_{\text{exp}} \approx \mathrm{d}r \sum_i y_i$. Owing to the assumption that each $\sigma_i \approx \sigma_0$, the relative error for $\langle e^{-Q}\rangle_{\text{exp}}$ is estimated as the sum, $\frac{\sigma_{\text{LHS}}}{\langle e^{-Q}\rangle_{\text{exp}}} = \mathrm{RE}(\text{norm}) + \mathrm{RE}(\sum_i y_i) + \mathrm{RE}(\mathrm{d}r)$. For the relative error $\mathrm{RE}(\sum_i y_i)$, we use a conservative estimate based on the prior analysis where χ^2 per degree of freedom equated to one as $\mathrm{RE}(\sum_i y_i) = \frac{\sqrt{N}\sigma_0}{\sum_i y_i}$. Note that the contribution to the error from $\mathrm{RE}(\sum_i y_i)$ would vanish in the case if the data is perfectly described by the theoretical model, i.e., when $y_i = y_i^0$.

SUPPLEMENTARY NOTE 6: COMPARISON FOR THE PROBABILITY DISTRIBUTION $p(Q)$ FROM REPEATED QUBIT MEASUREMENTS ON A SINGLE QUBIT

In the methods section of the main text, we briefly discussed how the probability distribution $p(Q)$ is computed from the experimental data for spin measurements with a uniformly initialized cloud. Here we detail the process. First, we compute the arrow of time $Q(r_i)$ for each of the data points r_i by using Equation (2) of the main text, and the corresponding probability densities $p(r_i)\mathrm{d}r$. Computing the arrow of time $Q(r_i)$ from Equation (2) of the main text requires that we square the measured probability $p(r_i)$, and divide by the determinant of the effect matrix, $M^\dagger(r)M(r)$, and then take a logarithm of this ratio. Note that the effect matrix does not depend on the initial spin of the system, and only depends on the measurement strength, which in turn depends on the separation between the identical Gaussian clouds, as well as the standard deviation of the Gaussian. Therefore the denominator can be thought of as a form factor for the measurement process. This simple dependency of $Q(r_i)$ on the measured quantities makes the LHS of the fluctuation theorem $\langle e^{-Q}\rangle$ straightforward to compute from the experimental data itself.

In addition, we are interested in knowing how Q is distributed in probability as this distribution contains useful information regarding the average arrow of time $\langle Q\rangle$, as well as shows instances where the arrow of time is estimated backward (negative Q). Such information is not immediately accessible from the probability distribution of the readout $p(r)$. In order to construct the probability distribution $p(Q)\mathrm{d}Q$ from $p(r)\mathrm{d}r$, we compute $Q(r)$ and $p(Q(r))$ for each r. In this step, it is possible that different r values may have similar Q, with different probability densities $p(r_1)\mathrm{d}r$ and $p(r_2)\mathrm{d}r$. Binning the probabilities of Q by ordering the list with increasing Q ensures that $p(Q)\mathrm{d}Q$ represents probability density summed over points r_1 and r_2 for a given Q within range dQ. The probability distribution $p(Q)$ shown in the main text as well as the supplement corresponds to binning nearest four values of $p(Q)$ with the corresponding average Q along the x axis. The probability distribution $p(Q)$ can also be computed analytically for the case when $z = 0$, given in Equation (3) of the main text. To demonstrate the consistency of our approach for other cases as well, in Supplementary Fig. 37, we have generated the probability distribution shown in Figure 2(c) of the main text assuming the equivalent measurement process on a single qubit, repeated many times to obtain the statistics.

SUPPLEMENTARY NOTE 7: VERIFYING THE FLUCTUATION THEOREM FOR THE EXPERIMENTAL DATA

The results in the main text pertaining to the fluctuation theorem represented in Figures 2(b,d) and Figure 3 provide evidence for the presence of absolute irreversibility in the quantum measurement process, where we compute the LHS of the fluctuation theorem $\langle e^{-Q}\rangle$ and show that it is a number $1-\mu$ between zero and one. A difficulty for the observed data we have is that μ appearing in the RHS of the fluctuation theorem cannot be estimated in a simple manner directly from the experimental data. If we look at the quantities appearing in the definition of μ given in Supplementary Equation (18), the denominator of the integrant is the forward probability density, which is directly measured, but the numerator depends not only on the model we assume for the measurement process, but also on the initial quantum state $|\psi\rangle$, as well as its orthogonal quantum state $|\bar{\psi}\rangle$. This additional dependency suggests that a direct method to estimate this quantity is therefore not straightforward from the experimental data we have. A different experimental configuration of our setup which may allow directly measuring μ independently of the LHS is also not immediately evident.

Regardless, we take a hybrid approach where we estimate the numerator based on the fit parameters we obtain and evaluate the denominator directly from the measured data, and evaluate the integral expression in Supplementary Equation (18) as a sum. A similar approach is taken for the Gaussian-Gaussian Stern–Gerlach experiment as well. In both cases, our estimate for μ deviates from the model predictions to some extent, while the same data provides good agreement for the LHS of the fluctuation theorem, providing excellent evidence for the existence of absolute irreversibility in the measurement process. We associate the better agreement of our methods to estimate the LHS of the fluctuation theorem to the nature of the quantity being computed. The measure $\langle e^{-Q}\rangle$ is similar to estimating the moment generating function $\langle e^{kQ}\rangle$ for the probability distribution at the value $k=-1$. Moment generating functions by definition contain information from all the higher order moments of the distribution as well; this therefore provides a more accurate prediction of the LHS. The RHS computed from Supplementary Equation (18) do not appear to have such an immediate correspondence, partially explaining why our methods give good predictions for the LHS of the fluctuation theorem. This is demonstrated in Supplementary Fig. 38, Supplementary Fig. 39, and Supplementary Fig. 41, where the error bars for μ is computed by following the same approach we detailed for computing the error bars in $\langle e^{-Q}\rangle_{\text{exp}}$. Supplementary Fig. 40 additionally shows the corresponding data set for one example, which indicates that for the same data set, the estimates summed over to obtain $\langle e^{-Q}\rangle_{\text{exp}}$ agrees rather well with the corresponding fit prediction, while the estimates summed over to obtain μ incur more errors.

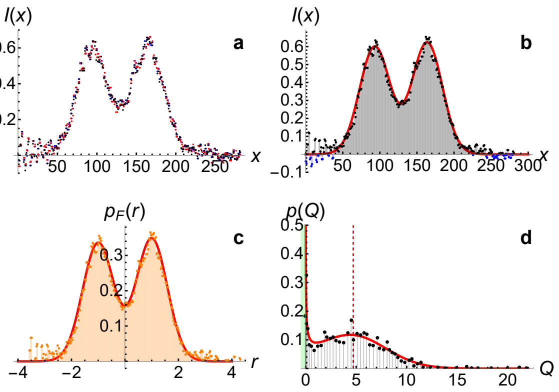

Supplementary Figure 5: (a) The raw data is constructed as the average of three nearby pixel values (shown in different colors) through the median of the cloud. (b) The raw data fit to a two mode Gaussian profile. The recognizable background noise (as negative points in the column density profile) are omitted in the data fit. (c) The raw data re-scaled to the readout variable r. (d) The corresponding arrow of time distribution $p(Q)$ for $z = 0.02,\ \sigma^2 = 0.34$.

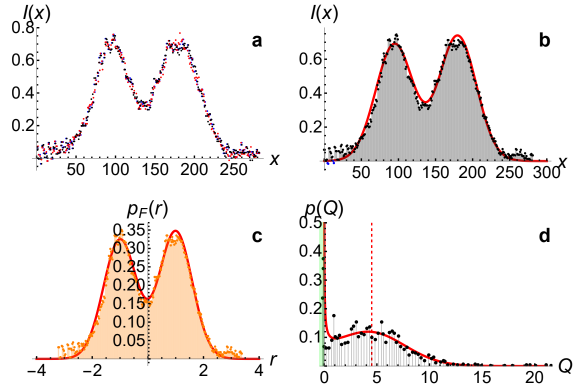

Supplementary Figure 6: $z = 0.03,\ \sigma^2 = 0.35$.

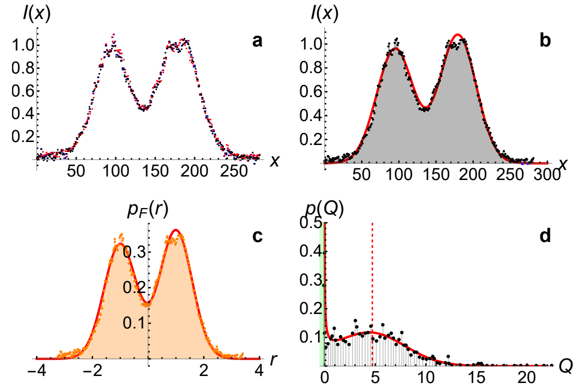

Supplementary Figure 7: $z = 0.06,\ \sigma^2 = 0.34$.

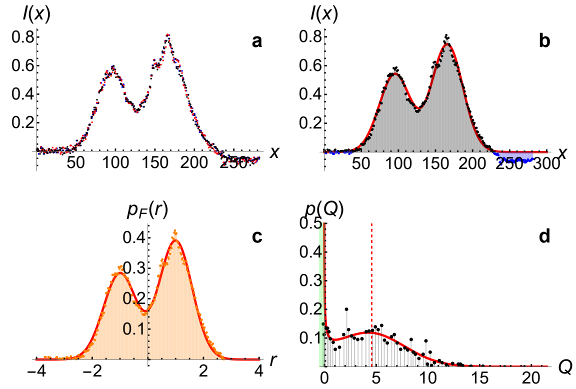

Supplementary Figure 8: $z = 0.16,\ \sigma^2 = 0.35$.

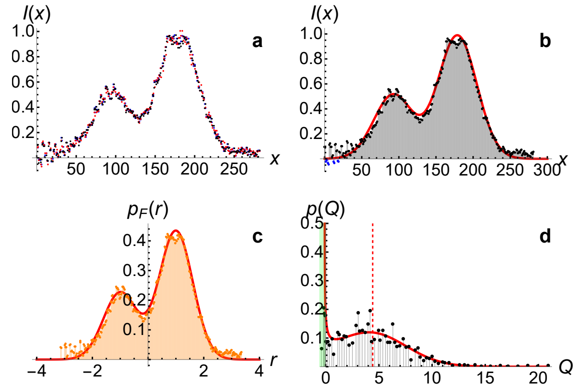

Supplementary Figure 9: $z = 0.32,\ \sigma^2 = 0.36$.

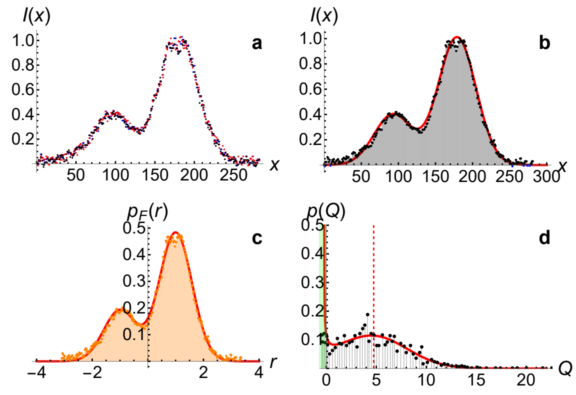

Supplementary Figure 10: $z = 0.43, \ \sigma^2 = 0.35$.

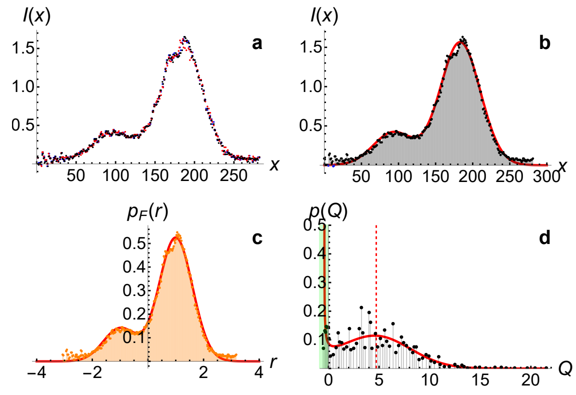

Supplementary Figure 11: $z = 0.58, \ \sigma^2 = 0.36$.

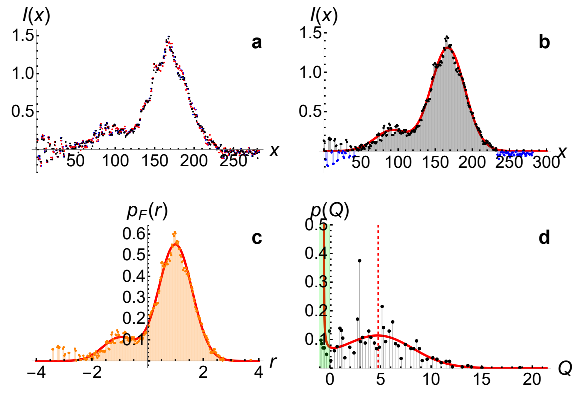

Supplementary Figure 12: $z = 0.67, \ \sigma^2 = 0.36$.

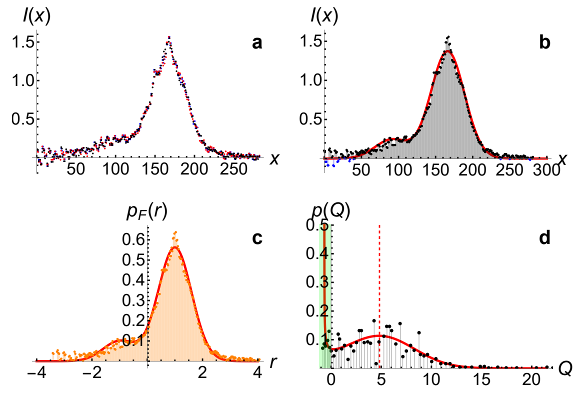

Supplementary Figure 13: $z = 0.7, \ \sigma^2 = 0.36$.

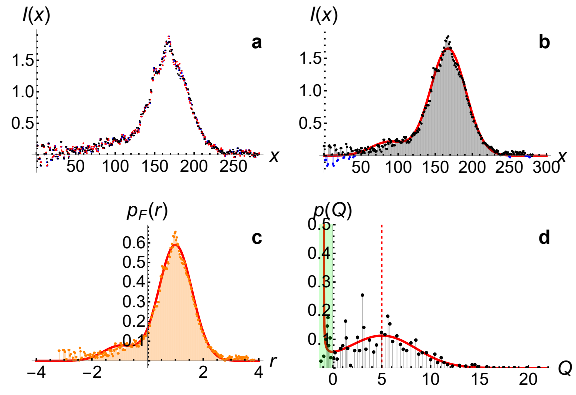

Supplementary Figure 14: $z = 0.77, \ \sigma^2 = 0.36$.

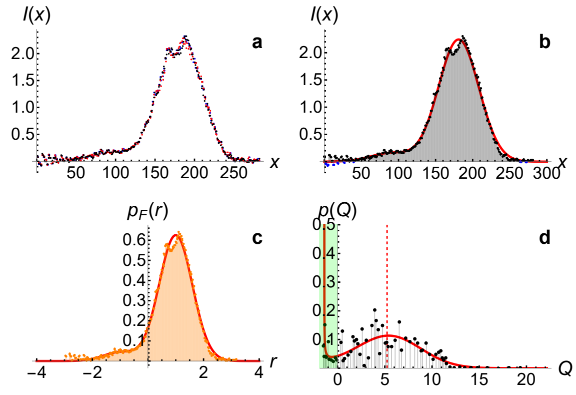

Supplementary Figure 15: $z = 0.87, \ \sigma^2 = 0.36$.

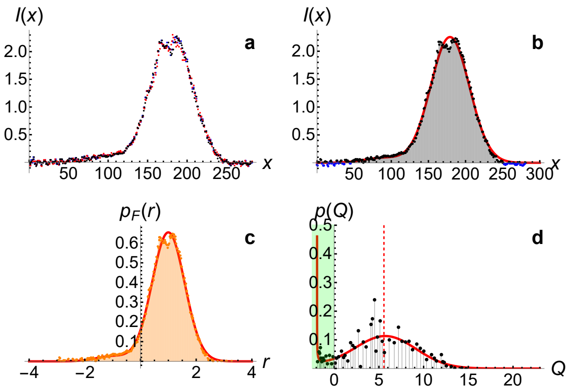

Supplementary Figure 16: $z = 0.92,\ \sigma^2 = 0.34$.

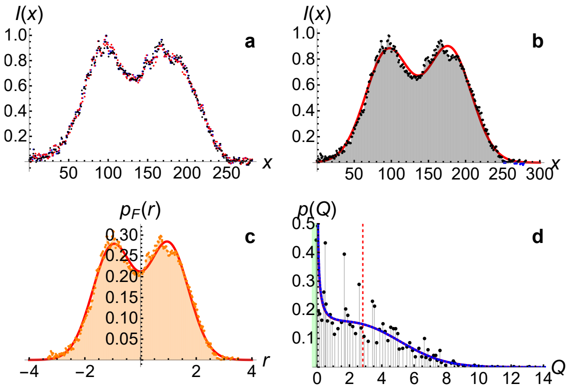

Supplementary Figure 17: (a) The raw data is constructed as the average of three nearby pixel values (shown in different colors) through the median of the cloud. (b) The raw data fit to a two mode Gaussian profile. The recognizable back ground noise (as negative points in the column density profile) are omitted in the data fit. (c) The raw data re-scaled to the readout variable r. (d) The corresponding arrow of time distribution $p(Q)$ for $z = 0.01,\ \sigma^2 = 0.53$. black profile indicate the theoretical prediction for $p(Q)$.

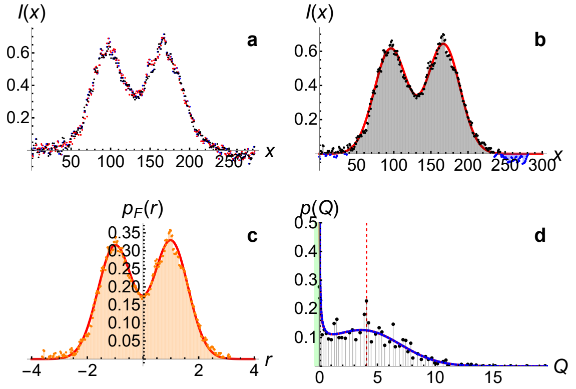

Supplementary Figure 18: $z = 0.02,\ \sigma^2 = 0.39$.

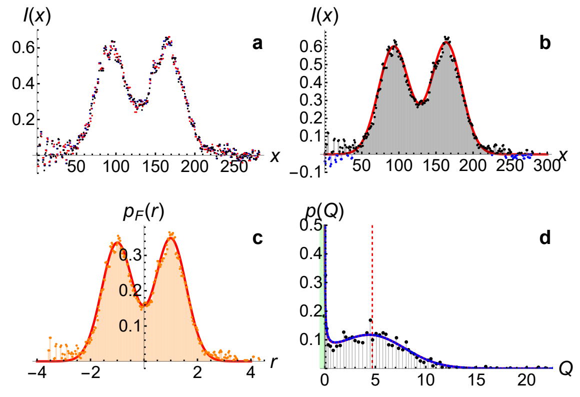

Supplementary Figure 19: $z = 0.02,\ \sigma^2 = 0.34$. (same data used for $\sigma^2 \simeq 0.35$ data set. Supplementary Fig. 5)

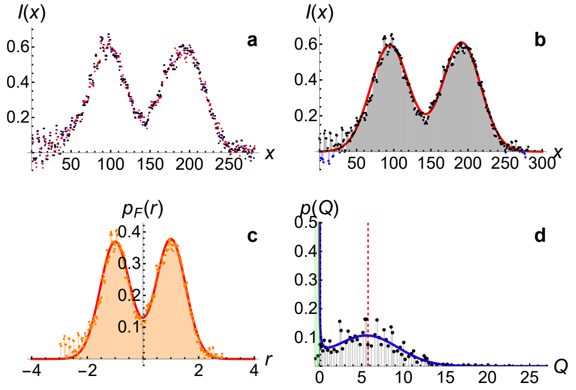

Supplementary Figure 20: $z = 0.01,\ \sigma^2 = 0.29$.

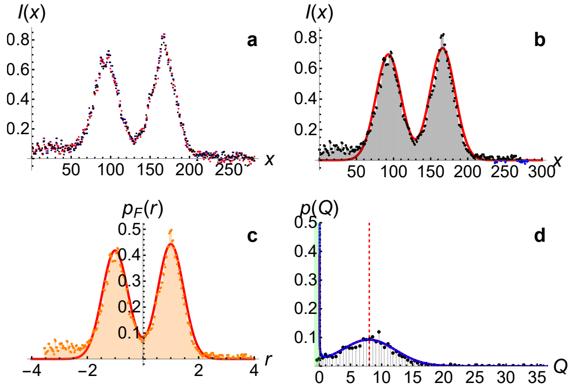

Supplementary Figure 21: $z = 0.03,\ \sigma^2 = 0.21$.

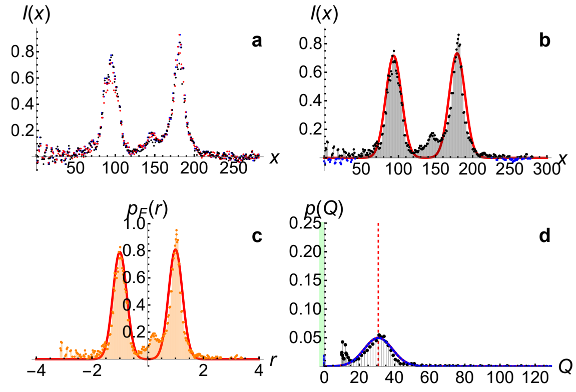

Supplementary Figure 22: $z = 0.01,\ \sigma^2 = 0.06$.

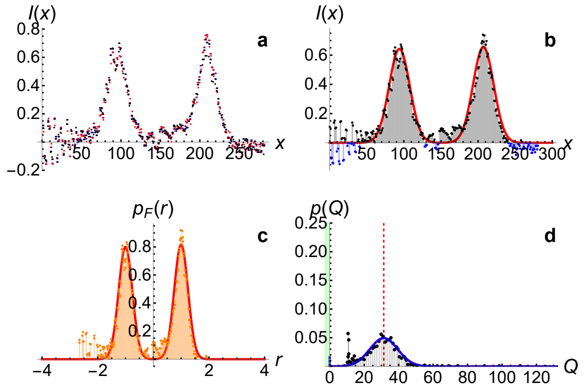

Supplementary Figure 23: $z = 0.01,\ \sigma^2 = 0.06$.

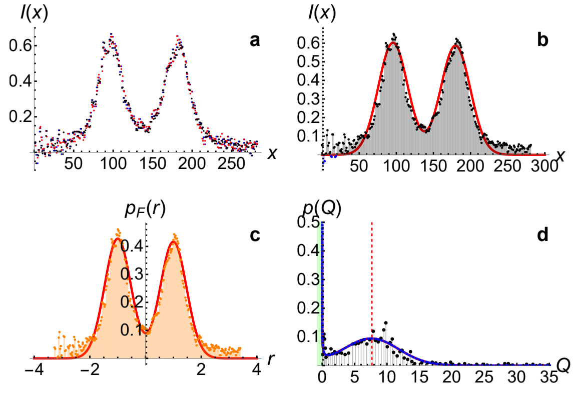

Supplementary Figure 24: $z = -0.01,\ \sigma^2 = 0.22$.

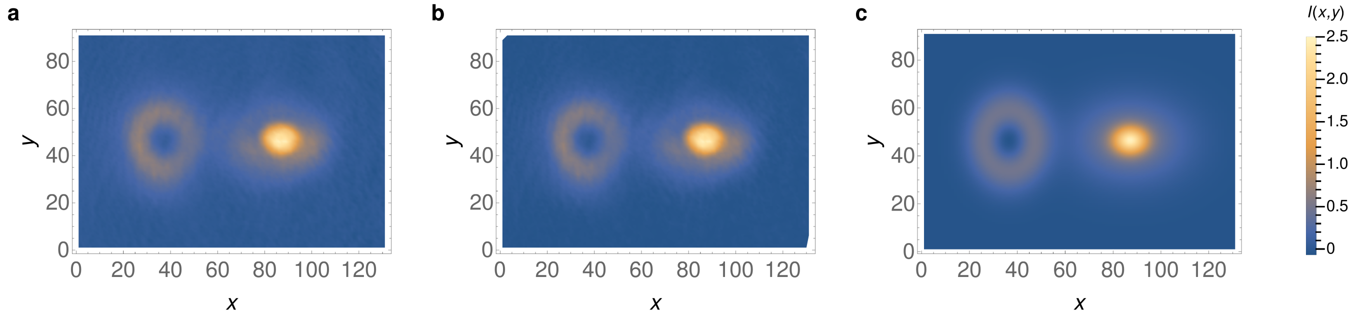

Fit parameters: σ_x = 16.2361, σ_y = 11.7227, σ_{x_0} = 4.70266, σ_{y_0} = 3.73971, w_x = 14.9383, w_y = 17.9806, x_{0G} = 87.2044, y_{0G} = 46.5161, x_{0LG} = 36.522, y_{0LG} = 46.3668, a = 0.609171, d = 0.773458

Supplementary Figure 25: (a) The raw data for column density of the atomic cloud obtained from the experiment in the LG-G case. The colorbar shows the full range of intensities in the raw data. The x and y axes are labeled in pixel units as obtained directly from the experiment. Pixel size in our experiment is $16\mu\text{m}\times16\mu\text{m}$, and the imaging system has unit magnification. (b) The raw data processed to only include pixels with measured positive values of cloud column density. Negative values are due to noise in the imaging process, see Supplementary Note 1. (c) Theory fit to the cloud column densities in (b), with fit parameters as shown below the figure.

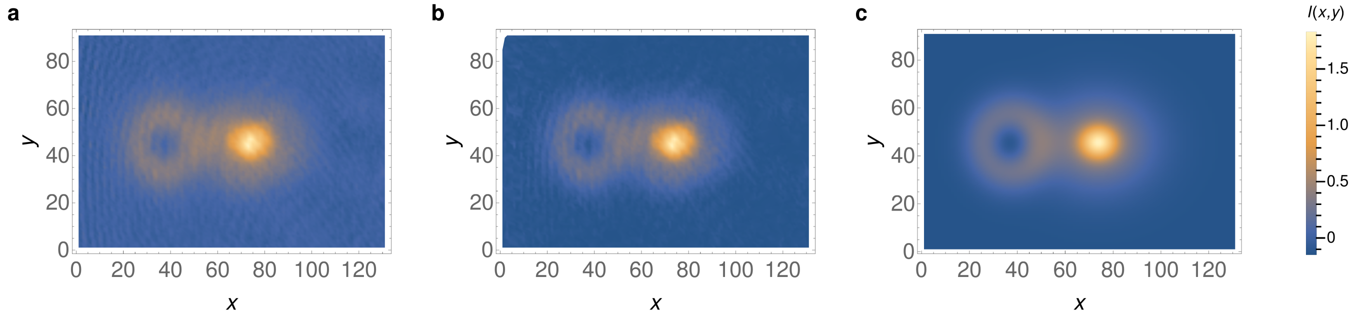

Fit parameters: σ_x = 15.7529, σ_y = 13.4495, σ_{x_0} = 4.8714, σ_{y_0} = 4.30411, w_x = 16.1488, w_y = 17.2611, x_{0G} = 73.8482, y_{0G} = 45.6189, x_{0LG} = 37.2159, y_{0LG} = 45.3709, a = 0.685018, d = 0.812306

Supplementary Figure 26

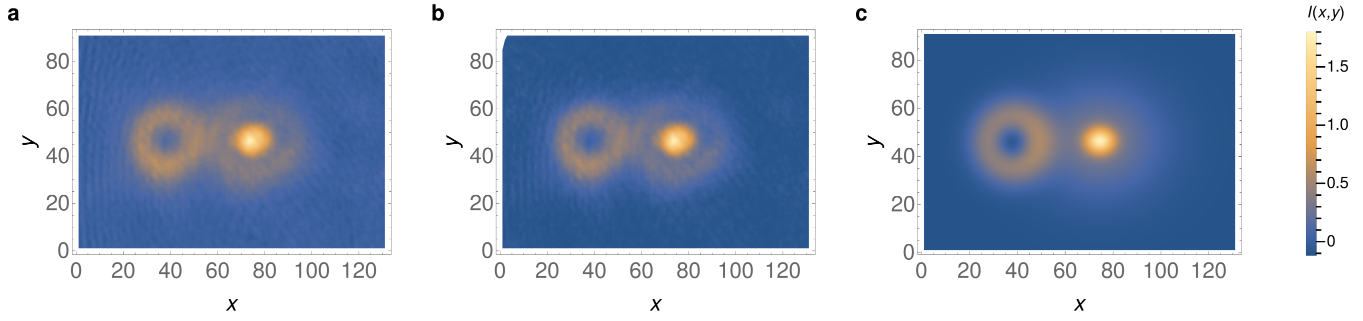

Fit parameters: σ_x = 17.8913, σ_y = 15.7676, σ_{x_0} = 4.1004, σ_{y_0} = 3.53301, w_x = 14.8188, w_y = 15.2747, x_{0G} = 74.5289, y_{0G} = 46.4832, x_{0LG} = 38.0435, y_{0LG} = 46.0378, a = 0.651131, d = 0.859567

Supplementary Figure 27

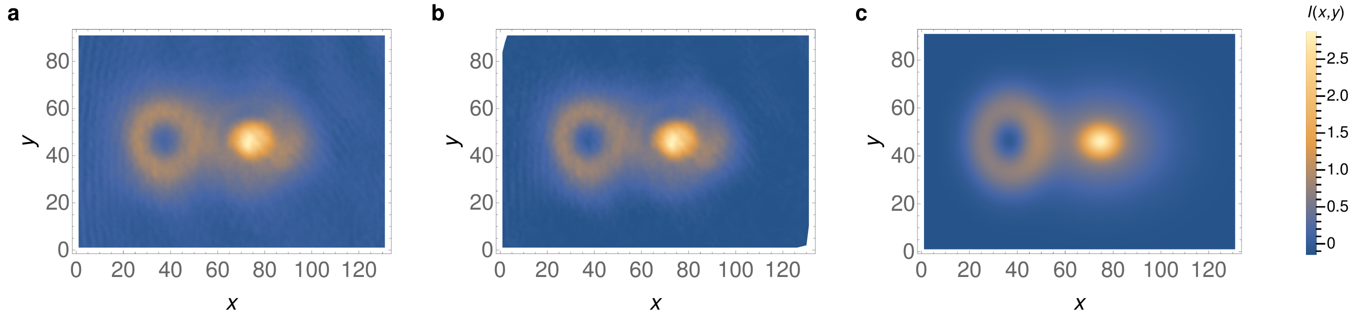

Fit parameters: $\sigma_x = 17.9485$, $\sigma_y = 14.065$, $\sigma_{x_0} = 4.90879$, $\sigma_{y_0} = 4.16059$, $w_x = 15.6839$, $w_y = 18.9367$, $x_{0G} = 74.731$, $y_{0G} = 46.1031$, $x_{0LG} = 36.909$, $y_{0LG} = 46.4305$, $a = 0.630159$, $d = 0.839511$

Supplementary Figure 28

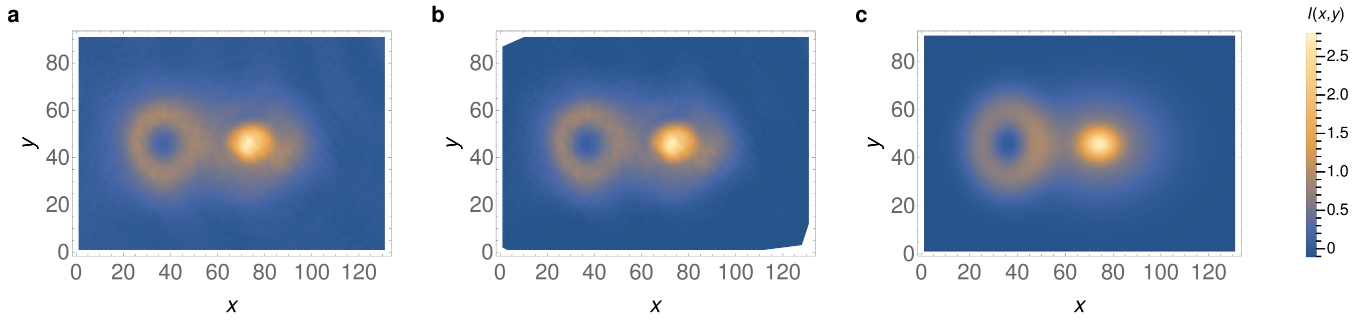

Fit parameters: $\sigma_x = 17.6385$, $\sigma_y = 14.0924$, $\sigma_{x_0} = 4.95733$, $\sigma_{y_0} = 4.16344$, $w_x = 15.8546$, $w_y = 18.929$, $x_{0G} = 74.224$, $y_{0G} = 45.7656$, $x_{0LG} = 36.4462$, $y_{0LG} = 45.882$, $a = 0.630495$, $d = 0.839978$

Supplementary Figure 29

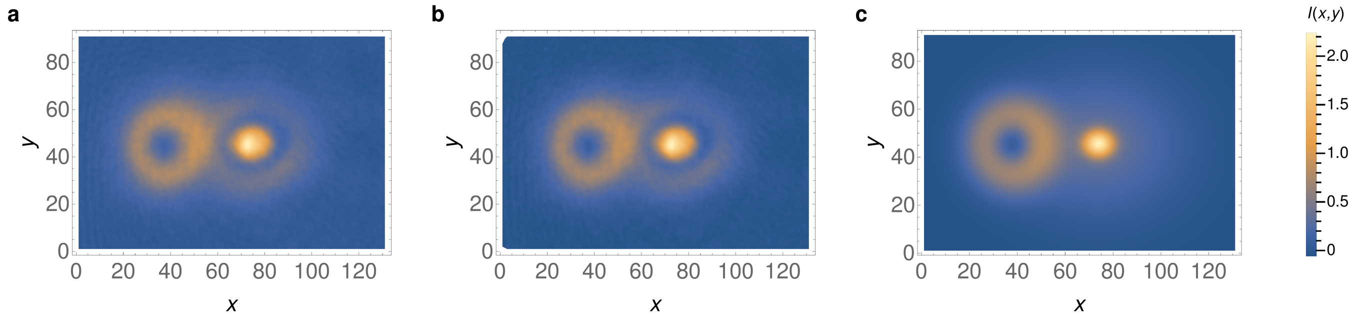

Fit parameters: $\sigma_x = 23.162$, $\sigma_y = 19.2639$, $\sigma_{x_0} = 4.19234$, $\sigma_{y_0} = 3.6674$, $w_x = 17.1464$, $w_y = 17.7641$, $x_{0G} = 73.9561$, $y_{0G} = 45.6306$, $x_{0LG} = 38.2106$, $y_{0LG} = 45.3858$, $a = 0.6135$, $d = 0.862371$

Supplementary Figure 30

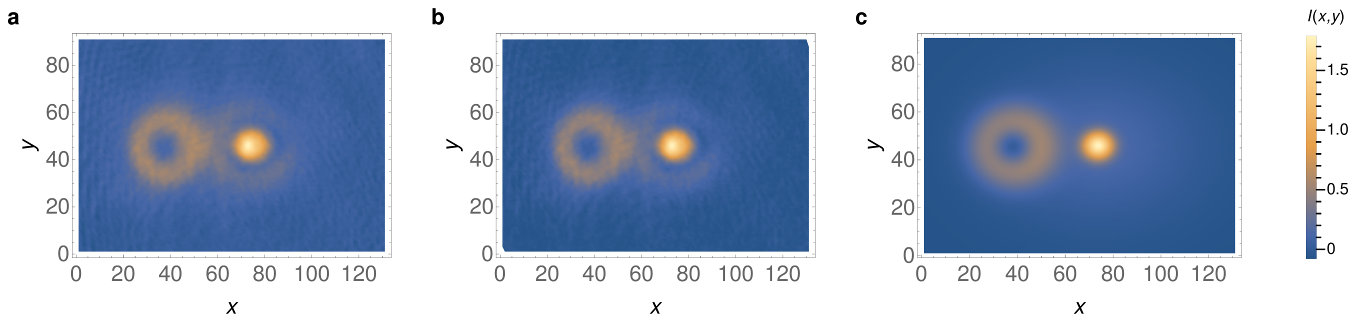

Fit parameters: σ_x = 28.5257, σ_y = 20.9682, σ_{x_0} = 4.18718, σ_{y_0} = 3.8159, w_x = 16.1836, w_y = 15.2603, x_{0G} = 73.7795, y_{0G} = 45.9856, x_{0LG} = 38.4233, y_{0LG} = 45.5461, a = 0.665874, d = 0.823315

Supplementary Figure 31

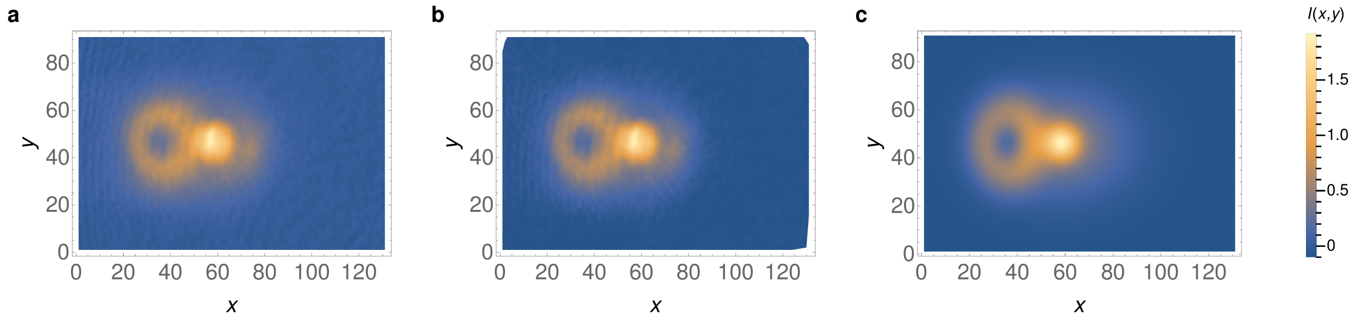

Fit parameters: σ_x = 18.8795, σ_y = 14.7319, σ_{x_0} = 4.93655, σ_{y_0} = 4.89444, w_x = 14.2096, w_y = 17.9659, x_{0G} = 58.6352, y_{0G} = 46.2876, x_{0LG} = 36.2114, y_{0LG} = 46.434, a = 0.677733, d = 0.812741

Supplementary Figure 32

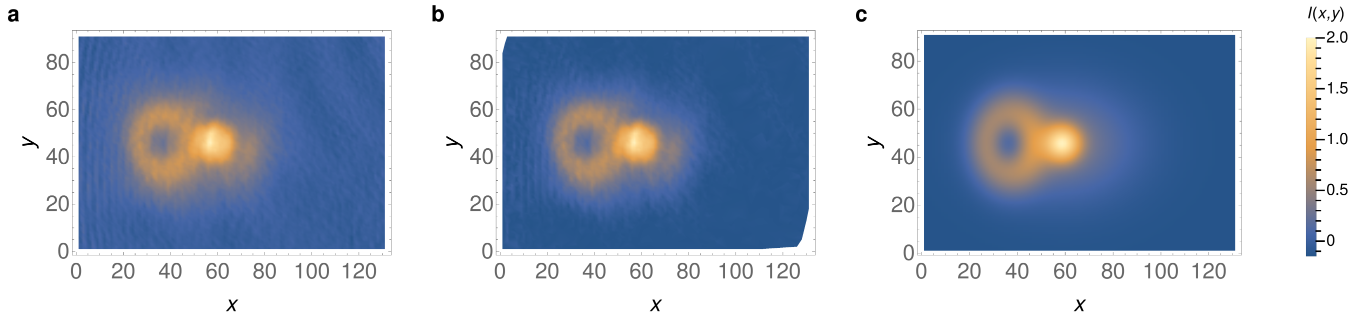

Fit parameters: σ_x = 20.6457, σ_y = 15.6456, σ_{x_0} = 4.99145, σ_{y_0} = 5.02796, w_x = 14.3477, w_y = 18.0665, x_{0G} = 59.0012, y_{0G} = 45.7655, x_{0LG} = 36.7643, y_{0LG} = 45.867, a = 0.678142, d = 0.809907

Supplementary Figure 33

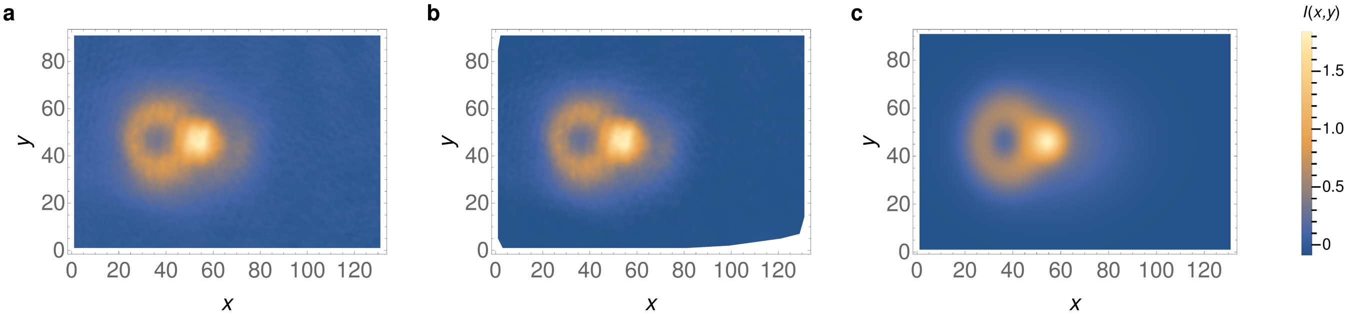

Fit parameters: σ_x = 19.6853, σ_y = 15.4006, σ_{x_0} = 4.50085, σ_{y_0} = 4.99885, w_x = 14.4632, w_y = 18.0088, x_{0G} = 55.0434, y_{0G} = 46.0862, x_{0LG} = 36.9386, y_{0LG} = 46.3954, a = 0.640849, d = 0.816258

Supplementary Figure 34

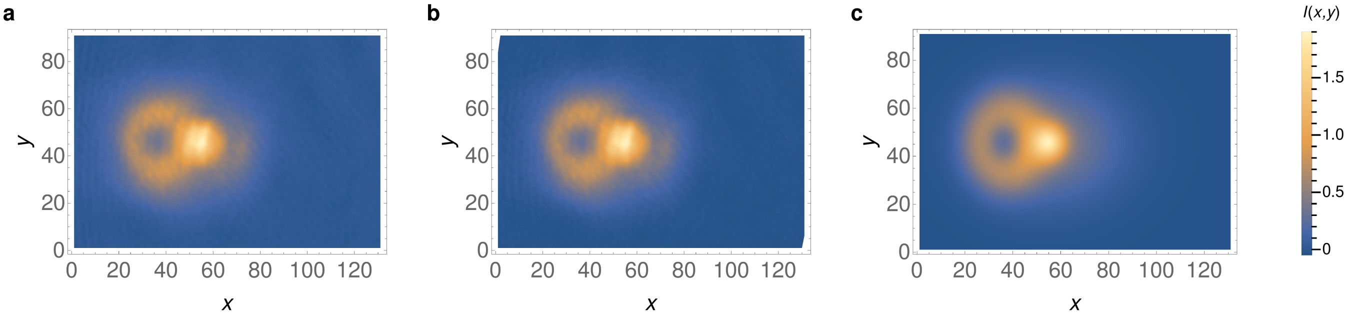

Fit parameters: σ_x = 20.175, σ_y = 16.278, σ_{x_0} = 4.69025, σ_{y_0} = 5.06608, w_x = 15.3772, w_y = 18.7143, x_{0G} = 55.3148, y_{0G} = 45.752, x_{0LG} = 36.8116, y_{0LG} = 45.8896, a = 0.660184, d = 0.852934

Supplementary Figure 35

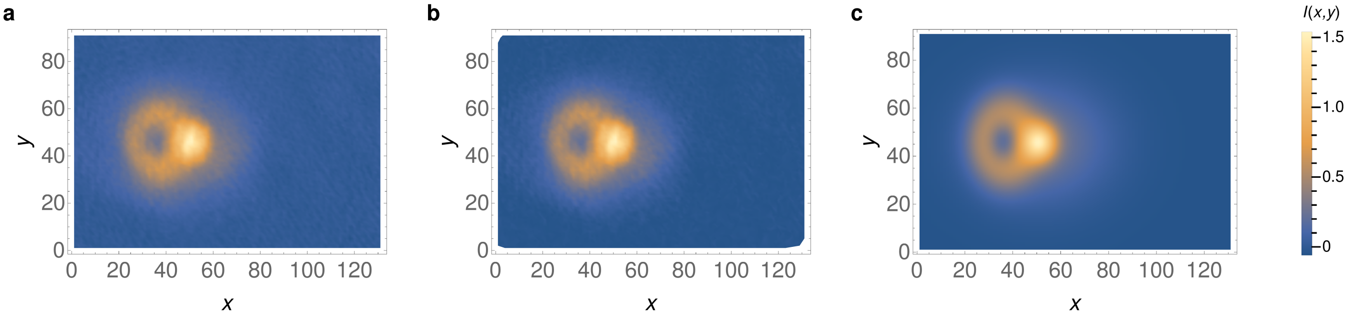

Fit parameters: σ_x = 19.6528, σ_y = 16.6505, σ_{x_0} = 4.64942, σ_{y_0} = 5.39275, w_x = 13.0914, w_y = 17.2809, x_{0G} = 51.5413, y_{0G} = 45.7238, x_{0LG} = 36.4234, y_{0LG} = 46.1642, a = 0.688569, d = 0.818632

Supplementary Figure 36

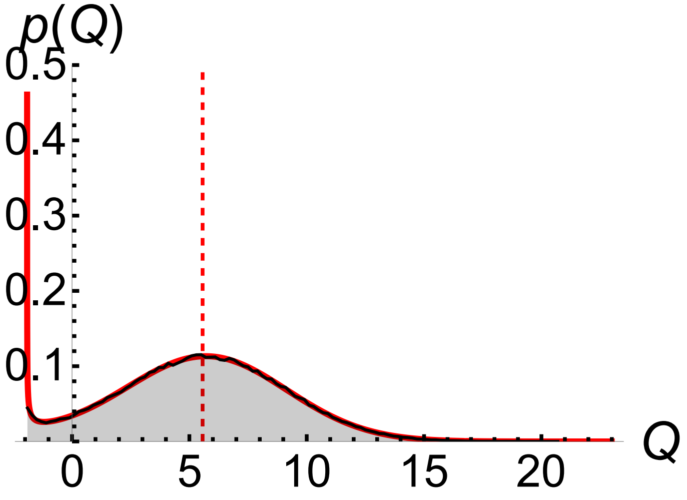

Supplementary Figure 37: The arrow of time distribution shown in this figure corresponds to Fig. 2(c) of the main text ($z \approx 0.92,\ \sigma^2 \approx 0.34$). The arrow of time distributions in the manuscript are constructed by employing a numerical change of variable $\{r, p(r)\} \rightarrow \{Q, p(Q)\}$. While the probability distribution is available in closed form from [9] for the case when $z = 0$, it is not straightforward to obtain this distribution for arbitrary z. Therefore, to demonstrate the validity of our numerical approach, we compare the distribution we obtain from numerical methods (red curve) to the probability distribution constructed from assuming measuring a single qubit for the same initial conditions many times (grey distribution) as typically done in superconducting qubit platforms [9, 11]. We find good agreement as shown above.

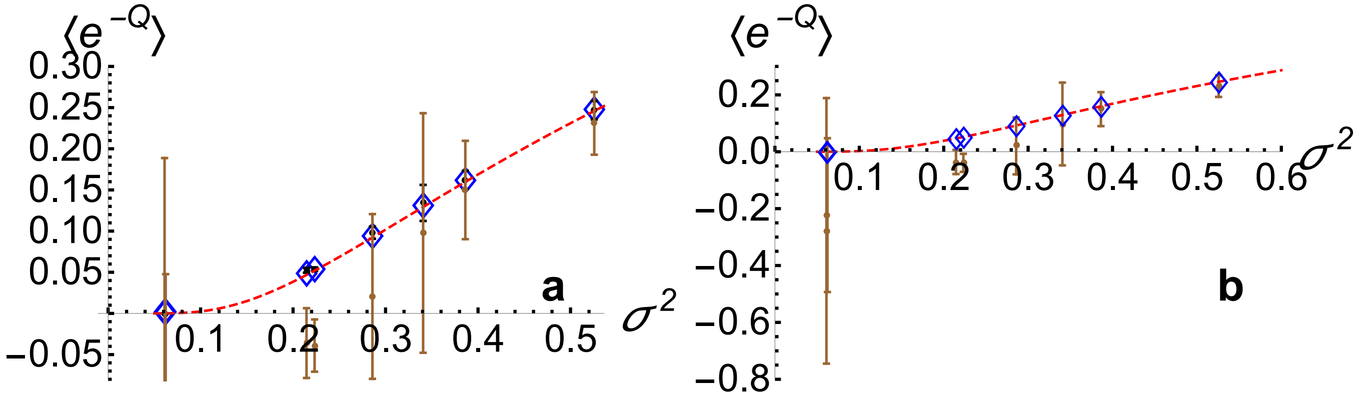

Supplementary Figure 38: (a) Our estimate for both sides of the fluctuation theorem $\langle e^{-Q} \rangle = 1 - \mu$ for Fig. 2(b) of main text. The RHS and associated error bars are shown in brown. (b) Showing the range of error bars for $1 - \mu$. Note that the center of the blue diamonds indicate the value of the corresponding theory prediction.

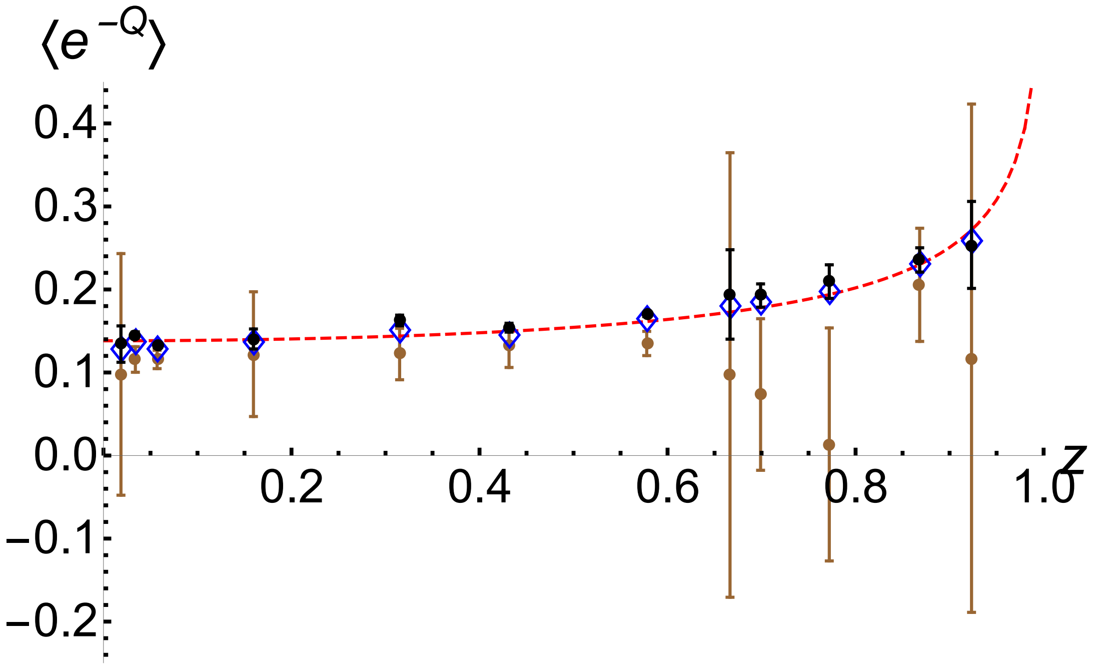

Supplementary Figure 39: Our estimate for both sides of the fluctuation theorem $\langle e^{-Q}\rangle = 1-\mu$ for Fig. 2(d) of main text. The RHS and associated error bars are shown in brown. Note that the center of the blue diamonds indicate the value of the corresponding theory prediction.

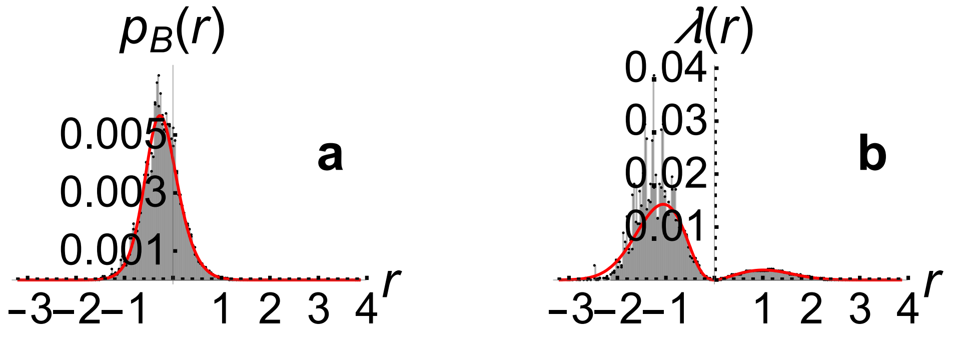

Supplementary Figure 40: Approach to estimating LHS and RHS of the fluctuation theorem. We define $\langle e^{-Q}\rangle = \int dr\, p_B(r)$ and $\mu = \int dr \lambda(r)$, where both λ and p_B are computed by including additional assumptions about the measurement process, such as the measurement is described by the Gaussian measurement operators in the Stern Gerlach experiment discussed in Fig. 2 of the main text. While we get good estimate for the LHS, $\langle e^{-Q}\rangle$, we note that the RHS computed in the same manner is not as efficient. This is because estimating $\langle e^{-Q}\rangle$ for a probability distribution makes use of information about all the higher order moments of the distribution (as it is like the moment generating function, $\langle e^{kQ}\rangle$ of the probability distribution $p(Q)$), while our method to estimate the RHS independently does not have such an immediate correspondence. This can be seen from the comparison to theory shown in (a) and (b) for one data point corresponding to Supplementary Fig. 14 ($z = 0.77, \sigma^2 = 0.36$).

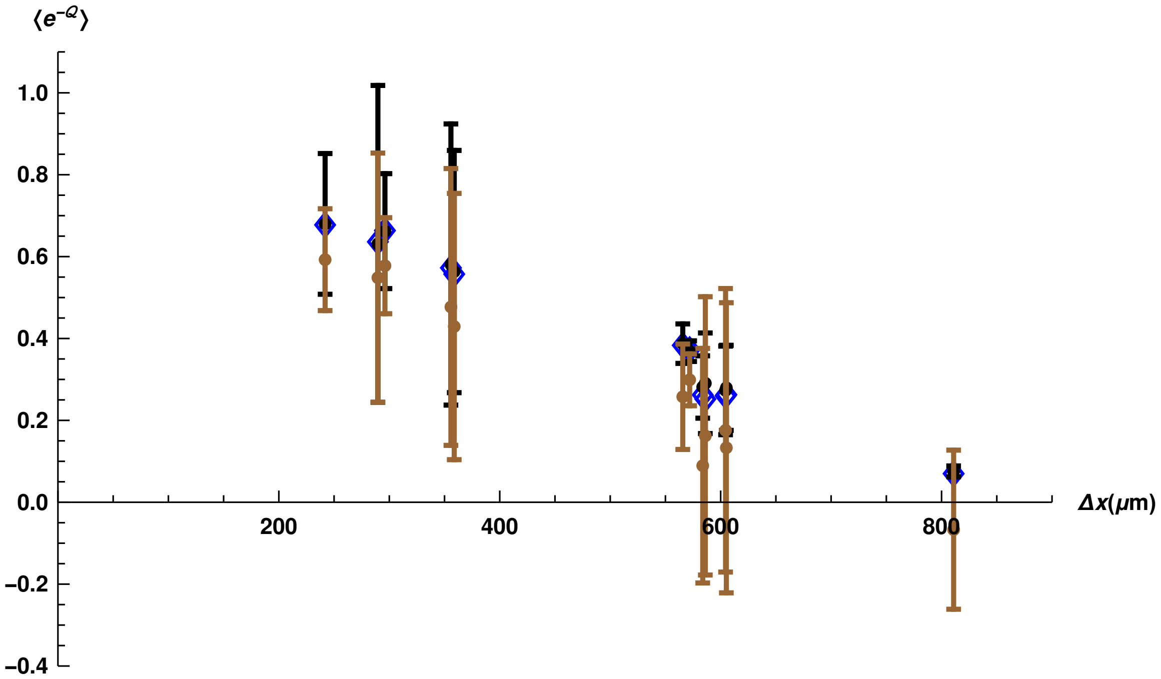

Supplementary Figure 41: Fluctuation theorem figure for the quantum many-body entangled wavefunction of the main text, where we show both the LHS and the RHS of the fluctutation theorem computed from experimental data. As in the spatially uniform case, we compute these quantities by assuming the form for the measurement operator discussed in the main text. We show (blue diamonds) prediction based on the theory fit and (black dots) prediction using experimental data for $\langle e^{-Q} \rangle$, and compare with (brown dots) prediction for $1 - \mu$ based on experimental data, computed from Supplementary Equation 18.

[1] Wright, K. C., Leslie, L. S., Hansen, A. & Bigelow, N. P. Sculpting the vortex state of a spinor BEC. *Phys. Rev. Lett.* **102**, 030405 (2009).

[2] Schultz, J. T., Hansen, A., Murphree, J. D., Jayaseelan, M. & Bigelow, N. P. Raman fingerprints on the Bloch sphere of a spinor Bose–Einstein condensate. *Journal of Modern Optics* **63**, 1759–1767 (2016).

[3] Schultz, J. T., Hansen, A., Murphree, J. D., Jayaseelan, M. & Bigelow, N. P. Creating full-Bloch Bose–Einstein condensates with Raman q-plates. *Journal of Optics* **18**, 064009 (2016).

[4] Schultz, J. T., Hansen, A. & Bigelow, N. P. A Raman waveplate for spinor Bose–Einstein condensates. *Optics letters* **39**, 4271–4273 (2014).

[5] Ketterle, W., Durfee, D. S. & Stamper-Kurn, D. M. Making, probing and understanding Bose–Einstein condensates (1999). cond-mat/9904034.

[6] Margalit, Y. *et al.* Analysis of a high-stability Stern–Gerlach spatial fringe interferometer. *New Journal of Physics* **21**, 073040 (2019).

[7] Manikandan, S. K., Elouard, C. & Jordan, A. N. Fluctuation theorems for continuous quantum measurements and absolute irreversibility. *Physical Review A* **99**, 022117 (2019).

[8] Manikandan, S. K. & Jordan, A. N. Time reversal symmetry of generalized quantum measurements with past and future boundary conditions. *Quantum studies: mathematics and foundations* **6**, 241–268 (2019).

[9] Dressel, J., Chantasri, A., Jordan, A. N. & Korotkov, A. N. Arrow of time for continuous quantum measurement. *Physical review letters* **119**, 220507 (2017).

[10] Young, P. Everything you wanted to know about data analysis and fitting but were afraid to ask. *arXiv preprint arXiv:1210.3781* (2012).

[11] Harrington, P., Tan, D., Naghiloo, M. & Murch, K. Characterizing a statistical arrow of time in quantum measurement dynamics. *Phys. Rev. Lett.* **123**, 020502 (2019).
